# Section on State Medicine

**Published:** 1881-06

**Authors:** 


FIRST DAY.

STATE MEDICINE.—Chairman, Dr. J. T. Reeve, of Wisconsin; Secretary, Dr. R. G. Jennings, of Little Rock, Ark.

The hour of meeting having arrived, the Secretary, Dr. R. G. Jennings, of Little Rock, Ark., called the Section to order.

A letter was read from the President, Dr. J. T. Reeve, of Wisconsin, regretting his inability to attend, both on account of ill-health and imperatively pressing engagements.

On motion of Dr. John S. Billings, U. S. A., the Section was adjourned until this evening at 3 o'clock.

SECOND DAY.

STATE MEDICINE.—This Section met at Wilkinson Hall, at 3 o'clock. Dr. J. S. Billings, U. S. A., Chairman *pro tem.;* Dr. R. G. Jennings, Secretary.

Dr. J. L. Cabell, of the University of Virginia, read an interesting paper on "The National Board of Health and the International Sanitary Conference of 1881."

Dr. Cabell alluded to the illiberal action of the National Congress in reference to the National Board of Health, and spoke of the success that had attended the efforts of the New York physicians for the establishment of a State Board of Health. He also gave a history of the establishment of the National Board, and the reasons why it had not secured sanitary regulations in States which had not established State Boards; and stated the reasons which impelled the calling of an International Sanitary Conference. Dr. Cabell proceeded to give a very interesting account of the proceedings of that Conference, and the good work accomplished by it. [This Conference, it will be remembered, met at Washington the latter part of January and the first of February last.]

After dwelling for some time upon the work prepared at the Conference, Dr. Cabell concluded as follows:

"There is, therefore, good reason for hoping that an international agreement may be arrived at between the States most frequently threatened with epidemic invasions. And, aside from this, the degree of attention which, as a result of the deliberations of the Conference, has been given to the subject of maritime sanitary police, cannot be without fruit in securing greater cleanliness, better ventilation of ships sailing on the high seas; and in general, an improved sanitary condition of these important instruments of commerce, which become so often the carriers of the most deadly contagion, from the failure to use such precautions as sanitary science suggests, and, as it is hoped, will now be enforced among the maritime powers of the world."

The paper was referred to the Committee on Publication.

Dr. C. F. Folsom, of Boston, read a paper on the relation of "The State to the Insane."

Dr. Folsom spoke of the difference in the treatment of the insane, adopted at this time from that which was formerly adopted. He also gave valuable statistical information as to the number of insane in various sections of the country. He argued in favor of the establishment of State Lunacy Boards. Among the points made were: First of all, that a lunacy board should embrace men with a thorough knowledge of insanity and its treatment. The chief duties of this board should be to secure proper care for the insane in private dwellings, where they are very much liable to neglect. Secondly, They should require the commitment of lunatics to the asylum by necessary copies of the commitment papers, and otherwise looking into the cases, so as to be able to tell whether the lunatic should be retained for care or be discharged.

The paper was in every respect an able one, and full of valuable information. Dr. Folsom was warmly congratulated, and his paper was referred to the Committee on Publication.

The paper was discussed in general commendation by Doctors Grissom, of North Carolina, and Hughes, of St. Louis.

Dr. Grissom made the point that if insanity is treated promptly, as other physical diseases are, that it is comparatively curable; but the custom is to delay action too long. One reason of this is that some people seem to think it disreputable to have an insane person in the family. When insanity is recognized as a purely physical disease and treated as such, more cures will be effected. The people must be educated to understand that to be insane is no cause for odium.

At the conclusion of these remarks, the Section adjourned until 3 o'clock, P. M.

STATE MEDICINE.—In consequence of the small attendance no meeting of this Section was held yesterday evening.

Papers on "Cellars," by Dr. A. N. Bell, of New York City, and on "The Necessity and Means of Removing Putrescible Matters from Inhabited Places," by Dr. O. W. Wright, of Milwaukee, Wisconsin, were received by the Secretary from the authors (who did not attend) and turned over to the Permanent Secretary.

